# High-Intensity Interval Training in Normobaric Hypoxia Improves Cardiorespiratory Fitness in Overweight Chinese Young Women

**DOI:** 10.3389/fphys.2017.00175

**Published:** 2017-03-23

**Authors:** Zhaowei Kong, Qingde Shi, Jinlei Nie, Tomas K. Tong, Lili Song, Longyan Yi, Yang Hu

**Affiliations:** ^1^Faculty of Education, University of MacauMacau, China; ^2^School of Physical Education and Sports, Macao Polytechnic InstituteMacau, China; ^3^Department of Physical Education, Dr. Stephen Hui Research Centre for Physical Recreation and Wellness, Hong Kong Baptist UniversityHong Kong, China; ^4^Sports Science Research Center, Beijing Sport UniversityBeijing, China

**Keywords:** normobaric hypoxia, high-intensity interval training, maximal oxygen uptake, blood lipids, leptin, body composition

## Abstract

Previous studies have investigated the effects of high-intensity interval training (HIIT) on cardiorespiratory fitness and body composition in overweight populations. However, the additive effect of HIIT and hypoxia on health parameters is not clear. This study compared the effects of HIIT under hypoxic conditions on cardiometabolic function with that under normoxia in overweight Chinese young women.

**Methods:** A double-blind randomized controlled experimental design was applied. Twenty-four sedentary overweight Chinese young women (weight: 68.8 ± 7.0 kg, BMI: 25.8 ± 2.3 kg·m^−2^) participated in the HIIT under either normoxia (NORM, *n* = 13, PIO_2_: 150 mmHg, FIO_2_: 0.21) or normobaric hypoxia (HYP, *n* = 11, PIO_2_: 117 mmHg, FIO_2_: 0.15) for 5 weeks. HIIT was composed of 60 repetitions of 8 s maximal cycling effort interspersed with 12-s recovery per day, for 4 days per week. Cardiorespiratory fitness [peak oxygen uptake ($\overset{\cdot }{\text{V}}$O_2peak_), and peak oxygen pulse (peak O_2_ pulse)], serum lipid profile [triglycerides (TG), total cholesterol (TC), high-density lipoprotein cholesterol (HDL-C), and low-density lipoprotein cholesterol (LDL-C)], and body composition (regional and whole-body), were assessed at pre- and post-intervention during the days beyond the self-reported menstrual phase of the participants. Habitual physical activity and diary behavior were maintained during the intervention period.

**Results:** With similar daily energy intake and physical activity, the increases in $\overset{\cdot }{\text{V}}$O_2peak_ [NORM: 0.26 ± 0.37 L·min^−1^ (+11.8%) vs. HYP: 0.54 ± 0.34 L·min^−1^ (+26.1%)] and peak O_2_ pulse (NORM: +13.4% vs. HYP: +25.9%) for HYP were twice-larger than for NORM (*p* < 0.05). Although the 5-wk HIIT led to significant improvements in the ratios of TC/HDL-C (*p* = 0.035) and TG/HDL-C (*p* = 0.027), no significant group effects were found on the serum variables. Further, no significant changes in body composition or serum fasting leptin were observed in either group.

**Conclusion:** 5-wk of HIIT improved cardiorespiratory fitness and blood lipids in overweight Chinese young females, while the additive effect of the HIIT under normobaric hypoxia solely enhanced cardiorespiratory fitness, but not body composition or serum lipid profile.

## Introduction

Physical activity has been regarded as the most important prevention against a variety of chronic health disorders including poor cardiorespiratory fitness, coronary heart disease, metabolic syndrome and obesity (Booth et al., 2012). Although an increase in physical activity and decrease in sedentary behaviors are recommended in the majority of individuals, physical activity has been shown to undergo a dramatic decline during the transition from late adolescence to early adulthood (Anderson et al., 2011; Kwan et al., 2012).

“Lack of time” is often cited as a key barrier to regular exercise participation (Trost et al., 2002). High-intensity interval training (HIIT), characterized as achieving over 80% of maximal heart rate (HR_max_) (Weston et al., 2014), has been shown to be a time-efficient paradigm to induce at least similar physiological adaptations associated with continuous moderate-intensity training (Gibala et al., 2006, 2014). It has been shown that 2–6 weeks of HIIT consisting of four to six 30-s “all-out” Wingate cycling tests separated by 4–4.5 min of recovery could improve cardiorespiratory fitness and associated metabolic function and body composition in athletes and sedentary individuals, as well as in patients with chronic diseases (Gibala et al., 2006; Rakobowchuk et al., 2008; Richards et al., 2010; Astorino et al., 2011; Gist et al., 2014; Hazell et al., 2014). However, the feasibility of cycling HIIT exercise is questionable, as such a strenuous protocol is hard to adhere to among sedentary individuals (Boutcher, 2011). This is partly due to the elicitation of antagonistic emotions during the repetition of “all-out” efforts (Saanijoki et al., 2015). As the training adaptations resulting from the low-volume HIIT effort are possibly exercise-mode specific (Rampinini et al., 2016), Trapp et al. (2008) have introduced an alternative cycling HIIT protocol which is rather brief, composed of 60 repetitions of 8-s of cycling effort interspersed with 12-s of recovery, and found that young sedentary women who participated in the specific HIIT for 15 weeks could markedly improve cardiorespiratory fitness, body composition and insulin resistance.

There is a general consensus that hypoxic training in athletes is an effective ergogenic aid to enhance the functional capacity of the cardiorespiratory system and associated endurance performance, and the intervention has been used for more than a half century. The ergogenic effect of hypoxic training was partly attributed to the improved blood perfusion level and resultant enhanced oxygen utilization in skeletal muscles (Faiss et al., 2013a). The underlying mechanisms for such beneficial adaptations may be associated to the compensatory vasodilation to match an increased oxygen demand at the muscular level; and the hypoxic training-induced changes in capillary-to-fiber ratio, fiber cross-sectional area, myoglobin content and oxidative enzyme activity in skeletal muscles which arise through the oxygen-sensing pathway (Vogt et al., 2001; Casey and Joyner, 2012; Faiss et al., 2013b). Moreover, hypoxic exercise augments sympathetic activation (Lippl et al., 2010) and the release of stress hormones (Shukla et al., 2005; Kayser and Verges, 2013), leading to an increase in energy expenditure (Workman and Basset, 2012) and appetite suppression (Kayser and Verges, 2013), and associated alteration of lipid metabolism (Haufe et al., 2008). Such specific physiological adaptations to hypoxic training in humans appear to be in favor of optimizing cardiometabolic function as well as body composition in sedentary individuals in comparison to those resulting from normoxic training. In our previous study, we demonstrated additive improvements in weight loss and hemodynamics following 4 weeks of intermittent hypoxic training in young obese individuals in comparison to their normoxic counterparts when the hypoxic level, training regimen, habitual dietary intake and physical activity were well controlled in a residential training camp (Kong et al., 2014). Our findings support the previous notion that hypoxia training may be an efficient strategy to tackle the health crisis of obesity and associated comorbidities (Urdampilleta et al., 2012; Kayser and Verges, 2013; Millet et al., 2016). However, the previous findings of intermittent hypoxic training-induced alterations in cardiorespiratory fitness and body composition, in spite of equivocal, were mainly resulted from moderate continuous training (Gatterer et al., 2015; Gonzalez-Muniesa et al., 2015). There is dearth of research investigating the effect of short-term, low-volume HIIT performed in hypoxia on cardiorespiratory and metabolic function in a sedentary population.

The present study was conducted, using a double-blind placebo-controlled design, to investigate whether a 5-wk HIIT regimen (60 repetitions of 8-s cycling effort interspersed with 12-s recovery, 4 sessions per week) carried out in normobaric hypoxia (HYP, FIO_2_ = 0.15) would improve cardiometabolic function in overweight young women in comparison to similar training in normoxia (NORM, FIO_2_ = 0.21).

## Materials and methods

### Subjects

This study was conducted in accordance with the declaration of Helsinki, and approved by the Ethical Committee of the University of Macau for the Use of Human and Animal Subjects in Research. Twenty-nine sedentary, overweight, but otherwise healthy Chinese young women were recruited based on the overweight criteria in the Asian-Pacific region recommended by the World Health Organization (WHO, 2000). The inclusion criteria were as follows: age between 18 and 30 years; body mass index (BMI) ≥ 23.0 kg·m^−2^; body fat (%BF) ≥ 30%; body weight that remained relatively constant (± 2 kg) in the past 3 months; residence at altitudes below 1,000 m; no prior experience of hypoxic training; no previous engagement in any structured exercise; non-smokers; not taking oral contraceptives or any medication known to influence body weight during the past 6 months. Following an explanation of the purpose and constraints of the study, all participants gave their written consent to participate.

### Study protocol

**Figure 1:**
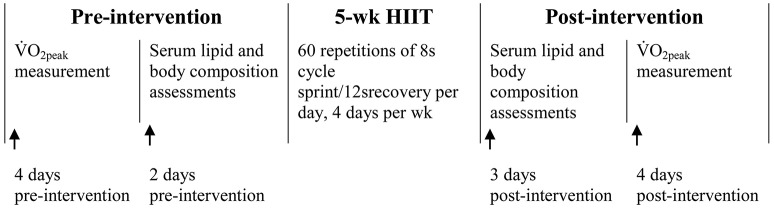
**The timeline of pre-and post-intervention measurements, as well as the 5-wk intervention of high-intensity interval training (HIIT)**.

Figure 1 shows the timeline of pre- and post-intervention measurements, as well as the 5-week HIIT intervention. Baseline measurements of body composition, cardiorespiratory fitness and fasting blood lipids were carried out 3–5 days before the intervention. Thereafter, participants with matching %BF and cardiorespiratory fitness were randomly assigned to either the NORM (*n* = 15) or the HYP (*n* = 14) HIIT group. The HIIT sessions were conducted in the 100 m^2^ hypoxic laboratory (Low Oxygen Systems GmbH, Germany) of the Sports Science Research Centre at Beijing Sport University. During the intervention, HIIT was performed at sea level (PIO_2_: 150 mmHg, FIO_2_: 0.21) for the NORM group or a simulated altitude of 2,500 m (PIO_2_: 117 mmHg, FIO_2_: 0.15) for the HYP group. The environment of the chamber remained constant during the intervention period with temperature of 22.2 ± 1.5°C and relative humidity of 21.9 ± 5.8%. In order to reduce the potential psychological influence, all participants and investigators were blinded to the group assignment and were not informed of the oxygen concentration in the chamber during the sessions. After intervention, measurements of body composition, cardiorespiratory fitness and fasting blood lipids, which were conducted in an identical manner to the baseline measurements, were carried out within 3–5 days following the last training session. All the pre- and post-intervention measurements were carried out during the days beyond the self-reported menstrual phase of the participants.

During the experimental period, all subjects were asked to maintain their daily activity and avoid altering their eating habits. During all testing and training sessions, a physician attended in case of emergency needs. At the end of the experiment, the awareness of group assignment was assessed by asking “Which condition do you think you trained in during the intervention?”

### High-intensity interval training

Accordingly to a previous study (Trapp et al., 2008), the HIIT protocol consisted of 60 repetitions of 8-s maximum effort on a Wingate testing bike ergometer (Monark 894 E, Sweden) interspersed with 12-s recovery with pedal frequency maintained between 20 and 30 rpm, 4 days per week, for 5 weeks. During a familiarization session prior to intervention, participants were requested to perform at least 10 8-s maximum efforts of the HIIT exercise, in order to ensure full understanding of the maximal involvement required during the intervention. The initial training workload was set at 1 kp for all participants. Once a participant was able to complete the 20-min full training protocol consistently at a given resistance, or demonstrated a significant decrease (≥ 5 bpm) in exercise heart rate (HR) in two consecutive sessions, the resistance was increased by 0.5 kp. During the HIIT, warm-up and cool-down exercises were standardized and identical in both groups. All participants exercised with carefully supervision. Polar HR monitor (Polar RS 800, Finland) and finger pulse oximeter (Nonin Onyx II Model 9560, USA) were used to monitor HR and peripheral oxygen saturation (SpO_2_), respectively, before, during and after each HIIT session. All the exercise data were concealed from participants until the completion of all experimental trials. Training load was reported via absolute and relative mean power output (MPO), percentage of HR_max_ (%HR_max_), and training impulse (TRIMP) as previously described (Banister, 1991).

### Physical activity and dietary assessments

Physical activity and dietary behavior outside the study were controlled and documented. Normal dietary and lifestyle habits were maintained as consistent as possible throughout the study. To record habitual activity level apart from the HIIT, each participant wore a pedometer (Ymax SW-200 digiwalker, Japan) 3 days (2 weekdays and 1 weekend day) per week during the week prior to and after the intervention, as well as during the 5-week HIIT intervention.

The diet of each participant was recorded on 3 days per week (2 weekdays and 1 weekend day) during the week prior to and after the intervention, as well as during the 5-week HIIT intervention, according to the guidelines of the Sports Nutrition Centre of the National Research Institute of Sports Medicine (NRISM) in China. The dietary records and corresponding energy intake were analyzed using the NRISM dietary and nutritional analysis system (version 3.1), designed for Chinese athletes and the general population. All participants were recommended to maintain a dietary macronutrient distribution of 60% carbohydrates, 35% fat and 15% protein.

### Graded exercise test

Participants performed a graded cycling test on the same bike ergometer (Monark 894 E, Sweden) 3 to 4 days pre- and post-intervention for measuring $\overset{\cdot }{\text{V}}$O_2peak_ via a breath-by-breath modular metabolic system (Max-II, AEI Technologies, PA, USA). Following a 3-min warm up, the graded cycling test began at 50 W and the power output increased by 25 W every 3 min until exhaustion. The plateau of oxygen uptake was used as the criterion for $\overset{\cdot }{\text{V}}$O_2peak_ despite increased workload, along with a respiratory exchange ratio of ≥1.1. The average of the highest $\overset{\cdot }{\text{V}}$O_2_ values for 15 consecutive seconds during the last stage was recorded as $\overset{\cdot }{\text{V}}$O_2peak_. HR was measured with Polar HR monitor (Polar F4M BLK, Finland) and the HR_max_ was defined as the highest value attained during the test. The peak O_2_ pulse was calculated as the $\overset{\cdot }{\text{V}}$O_2peak_ divided by the maximal HR and expressed as ml·beat^−1^.

### Serum profiles

Participants reported to the laboratory in the morning (7:00 a.m.) after a minimum 12 h overnight fast. Subjects were asked to refrain from physical exercise and alcohol ingestion for at least 48 h before each session. Following 30 min of rest on a chair, 8 mL venous blood from the antecubital fossa was collected. After static clotting and centrifugation, serum samples were removed and stored at −80°C until later analysis.

Serum lipids, including high-density lipoprotein cholesterol (HDL-C), low-density lipoprotein cholesterol (LDL-C), total cholesterol (TC) and total triglycerides (TG), were measured using an automatic biochemical analyzer (Olympus AU400, Japan). The intra-assay coefficients of variation (CV) for blood lipid assays were all within 5.0% (Deyi Biomedical Technology Co., Ltd., Beijing, China). Serum leptin was measured by a commercial enzyme-linked immunosorbent assay kit (Abcam, Cambridge, UK). The lower detection limits and intra-assay CVs were 5.3 pg·ml^−1^ and 2.4% for leptin, respectively (KingMed Center for Clinical Laboratory Co. Ltd, Guangzhou, China).

### Body composition measurements

Height was determined using standard methods with a stadiometer to the nearest 0.1 cm. Whole-body and regional fat mass and lean mass measurements were carried out 30 min following the venous blood collection. Subjects were scanned in a supine position by a dual-energy X-ray absorptiometry scanner (Norland XR-36 DXA densitometer, Norland Corporation, Fort Atkinson, WS, USA) and images analyzed using dedicated software (3.7.4/2.1.0; Norland Corporation). Lean mass and fat mass were calculated from the total and regional analysis of the whole body scan. The instrument was calibrated daily with the phantoms provided by the manufacturer.

### Statistical analysis

Statistical analysis was carried out using SPSS software (Version 20.0, IBM, New York, USA). All variables were checked by parametric statistics regarding conformation to a normal distribution by the Kolmogorov-Smirnov test. Independent sample *t*-tests were performed to determine the differences in training parameters and SpO_2_ between the two groups. A two-way repeated measures analysis of variance (group × time) was used to determine differences in pre- and post-training variables. Significant effects were subsequently analyzed using the Tukey *post hoc* test. As effect size measures of the main effect and the interaction effect, *partial* η^2^ was considered small if η^2^ < 0.01 and large if η^2^ > 0.14. Pearson product-moment correlation coefficients were computed to examine the relationships between variables. The sample size of subjects estimated for the power of 0.8 to detect a significant increase after HIIT was 8–12 for the primary outcome of $\overset{\cdot }{\text{V}}$O_2peak_. All tests for statistical significance were standardized at an alpha level of *p* ≤ 0.05, and all results were expressed as mean ± standard deviation.

## Results

### High-intensity interval training

During the intervention, two participants in NORM and three in HYP withdrew from the HIIT intervention for personal reasons that were not exercise-related. Accordingly, the data from 13 participants in the NORM group, and 11 subjects in the HYP group were valid for subsequent analyses. Among the participants who completed the study, compliance with the exercise intervention was 100% in the NORM and HYP groups. Eleven (46%) of the 24 participants provided an incorrect answer about the group assignment during the intervention period suggesting that the participants were blinded to the inhaled oxygen concentration during their exercise. No adverse events were reported during testing or training in either group.

**Figure 2:**
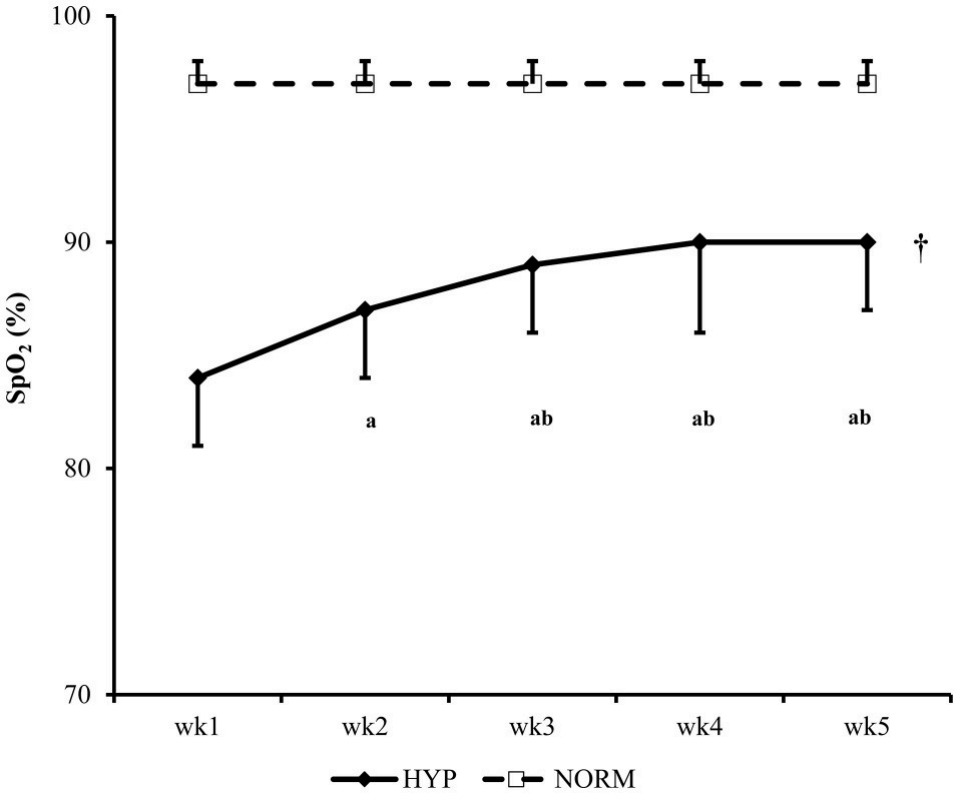
**The changes in peripheral oxygen saturation (SpO_2_) of participants during the 5-wk HIIT under normoxia (NORM) and normobaric hypoxia (HYP) are shown [partial η^2^ = 0.849 (time); 0.823 (group); 0.780 (time × group)]**. ^†^Significantly different from the corresponding values of the NORM group (*p* < 0.05). ^a^Significantly different from the wk1 value of the HYP group (*p* < 0.05); ^b^Significantly different from the wk 2 value of the HYP group (*p* < 0.05); HIIT, high-intensity interval training.

For the SpO_2_ of NORM and HYP groups, the main effects of time (*p* < 0.001, partial η^2^ = 0.849), group (*p* < 0.001, partial η^2^ = 0.823) and interaction (*p* < 0.001, partial η^2^ = 0.780) effects were significant. Throughout the 5-week HIIT intervention, SpO_2_ remained constant in the NORM group, while it increased progressively in the HYP group in the first 2 weeks, and plateaued in the subsequent weeks. The SpO_2_ of the NORM group in each week was significantly higher than the corresponding value of the HYP group (*p* < 0.05) (Figure 2).

Table 1 shows the HIIT details of NORM and HYP groups in each week during the intervention. During the 5-wk HIIT, the average exercise duration reduced progressively (*p* < 0.05), and the difference in the reduction of the exercise duration between the NORM and HYP groups was not significant (*p* > 0.05). In contrast, the average workload increased progressively across the 5-wk HIIT (*p* < 0.05) without differing between groups (*p* > 0.05). Likewise, the MPO of NORM and HYP groups were increased in similar magnitude, except during the 5th week, in which the MPO of NORM was slightly higher than that of HYP group (*p* < 0.05). For the TRIMP and % HR_max_, slightly reductions were observed across the 5-wk HIIT in both groups (*p* < 0.05), while the changes in the two variables of the HIIT were not significant (*p* > 0.05).

**Table 1:** **Training parameters of participants of the 5-wk high-intensity interval training regimen under normoxia (NORM) and normobaric hypoxia (HYP)**.

		**NORM**	**HYP**	**Time effect**		**Group effect**		**Time×group effect**	
				***p* partial η^2^**		***p* partial η^2^**		***p* partial η^2^**	
Workload (kp)				<0.001	0.964	0.270	0.055	0.220	0.250
	wk1	1.2±0.1	1.1±0.1						
	wk2	2.1±0.3^a^	2.0±0.3^a^						
	wk3	2.8±0.4^a^	2.6±0.6^a^						
	wk4	3.3±0.5^a^	2.9±0.6^a^						
	wk5	3.5±0.6^a^	3.5±0.3^a^						
Duration (min)				<0.001	0.676	0.521	0.019	0.208	0.256
	wk1	19.5±0.9	18.1±2.6						
	wk2	19.0±1.1	19.3±1.0						
	wk3	18.3±1.7	18.5±1.6						
	wk4	15.8±3.1^a^	16.1±2.8						
	wk5	16.6±2.1^a^	15.9±1.8						
MPO (W·kg^−1^)				<0.001	0.964	0.049	0.165	0.216	0.251
	wk1	2.1±0.5	1.9±0.3						
	wk2	2.8±0.5^a^	2.5±0.3^a^						
	wk3	3.4±0.6^a^	3.0±0.4^a^						
	wk4	3.6±0.8^a^	3.2±0.3^a^						
	wk5	3.9±0.6^a^	3.3±0.3^†^^a^						
% HR_max_				0.039	0.396	0.471	0.024	0.702	0.104
	wk1	86.2±4.0	88.4±5.2						
	wk2	86.5±4.6	87.5±5.1						
	wk3	86.4±3.4	86.8±5.4						
	wk4	86.3±2.9	86.8±4.8						
	wk5	84.3±3.0^a^	85.7±4.3						
TRIMP (au)				<0.001	0.706	0.738	0.005	0.757	0.090
	wk1	43.5±9.8	43.1±9.5						
	wk2	45.4±9.9	48.5±10.1						
	wk3	42.9±7.1	44.9±10.2						
	wk4	36.5±8.2	37.7±10.1						
	wk5	33.8±8.1^a^	32.0±8.3^a^						

*Values are expressed as means ± standard deviation. MPO, mean power output relative to body weight; %HR_max_, percentage of maximal HR; TRIMP (au), training impulse (arbitrary unit)*.

† *Significantly different from the corresponding values of the NORM group (p < 0.05)*.

a *Significantly different from the corresponding wk1 value in NORM and HYP group (p < 0.05)*.

### Habitual energy intake and activity level

The habitual energy intake and activity level of the participants during the week prior to and after the intervention, as well as during the 5-week HIIT intervention, are depicted in Table 2. No significant difference was noted within and between groups before, during and after intervention for habitual energy intake (*p* > 0.05); similar results were also found in the estimated habitual physical activity level (*p* > 0.05).

**Table 2:** **Habitual energy intake and physical activity level during the 1-week prior to the intervention (Pre), the 5 weeks of the intervention, and the 1 week after the intervention (Post) in NORM and HYP groups**.

		**NORM**	**HYP**	**Time effect**		**Group effect**		**Time×group effect**	
				***p* partial η^2^**		***p* partial η^2^**		***p* partial η^2^**	
Energy intake (kcal·day^−1^)				0.901	0.122	0.802	0.030	0.056	0.518
	Pre	2065 ± 582	2332 ± 869						
	wk1	2200 ± 856	2150 ± 713						
	wk2	2374 ± 918	2109 ± 703						
	wk3	1857 ± 644	2327 ± 811						
	wk4	2168 ± 889	1914 ± 431						
	wk5	1930 ± 634	2290 ± 561						
	Post	2175 ± 990	2110 ± 866						
Physical activity (steps·day^−1^)				0.131	0.425	0.173	0.087	0.386	0.299
	Pre	6983 ± 3655	7047 ± 3714						
	wk1	8797 ± 3776	6534 ± 3194						
	wk2	7938 ± 5782	8501 ± 3493						
	wk3	7283 ± 2787	7938 ± 5782						
	wk4	7056 ± 3489	7298 ± 4824						
	wk5	7500 ± 3180	6463 ± 3125						
	Post	6896 ± 3739	5663 ± 2674						

*Values are expressed as means ± standard deviation. NORM, high-intensity interval training in normoxia; HYP, high-intensity interval training in normobaric hypoxia*.

### Cardiorespiratory fitness, body composition and serum profile

**Figure 3:**
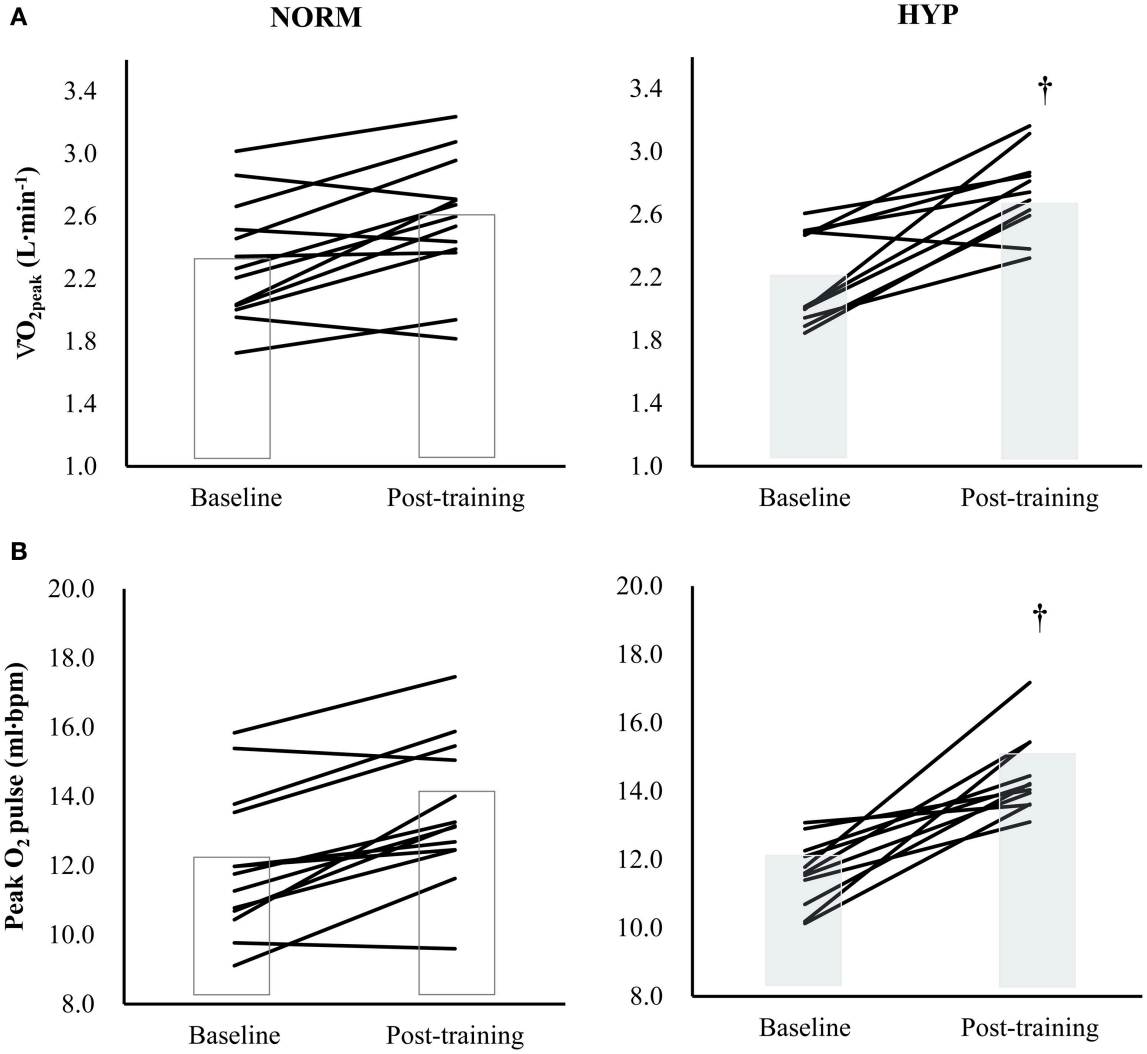
**The changes in (A)** $\overset{\cdot }{\text{V}}$O_2peak_[partial η^2^ = 0.626 (time); 0.144 (group); 0.177 (time × group)] and **(B)** Peak O_2_ pulse [partial η^2^ = 0.736 (time); 0.229 (group); 0.216 (time × group)] in individual participants (solid lines) and groups (bars) after 5-wk HIIT under normoxia (NORM, *n* = 13) and normobaric hypoxia (HYP, *n* = 11). ^†^Significantly different from corresponding NORM value (*p* < 0.05); HIIT: high-intensity interval training.

Table 3 shows the variables of cardiorespiratory fitness, body composition and serum lipid profile pre- and post-intervention. The significant main effect of time in absolute and relative $\overset{\cdot }{\text{V}}$O_2peak_ and peak O_2_ pulse (*p* < 0.001) suggested that there were significant increases in these three variables in both NORM and HYP groups. The significant interaction effect (*p* ≤ 0.05) further revealed that the increases in these three variables in the NORM group were significantly lesser in comparison to corresponding variables in the HYP group (Figure 3). Furthermore, the increase in time for which the participants sustained the exercise at the intensity corresponding to ≥ 90% of their HR_max_ between the last and first training session (NORM: 4.4 ± 3.6 min vs. 7.2 ± 8.3 min, HYP: 4.1 ± 3.4 min vs. 7.9 ± 8.8 min, *p* < 0.05) was positively correlated to the increase in absolute $\overset{\cdot }{\text{V}}$O_2peak_ (*r* = 0.55, *n* = 24, *p* < 0.01).

**Table 3:** **Cardiorespiratory fitness, body composition and serum lipid profile prior to (Pre) and after the intervention (Post) in NORM and HYP groups**.

	**NORM**		**HYP**		**Time effect**		**Group effect**		**Time×group effect**	
	**Pre**	**Post**	**Pre**	**Post**	***p* partial η^2^**		***p* partial η^2^**		***p* partial η^2^**	
Height (cm)	162.8±4.6		163.7±4.6							
Weight (kg)	68.2±8.1	68.4±8.3	69.5±5.8	69.6±6.2	0.795	0.003	0.981	0.000	0.902	0.001
BMI (kg·m^−2^)	25.7±2.2	25.8±2.1	26.0±2.4	26.0±2.6	0.815	0.003	0.507	0.022	0.941	0.000
$\overset{\cdot }{\text{V}}$O_2peak_ (L·min^−1^)	2.3±0.4	2.6±0.4	2.2±0.3	2.7±0.3	<0.001	0.626	0.081	0.144	0.041	0.177
$\overset{\cdot }{\text{V}}$O_2peak_ (ml·kg^−1^.min^−1^)	34.4±6.0	38.0±7.1	32.1±4.3	39.7±4.8	<0.001	0.604	0.143	0.104	0.050	0.158
Peak O_2_pulse (ml·beat^−1^)	12.5±2.2	14.1±2.0	12.1±1.1	15.2±1.3	<0.001	0.736	0.024	0.229	0.022	0.216
Total LM (kg)	41.3±4.5	41.1±4.8	41.5±2.9	41.4±3.3	0.594	0.013	0.651	0.010	0.925	0.000
Total FM (kg)	25.4±5.5	25.6±5.8	26.5±3.6	26.5±4.3	0.779	0.004	0.608	0.013	0.763	0.004
Total BF (%)	37.0±4.8	37.2±5.3	38.0±2.3	37.9±3.5	0.911	0.001	0.720	0.007	0.723	0.006
Trunk LM (kg)	18.1±2.4	18.1±2.6	18.3±1.6	18.0±1.6	0.297	0.049	0.239	0.069	0.418	0.030
Trunk FM (kg)	13.0±2.8	12.9±3.1	13.7±2.2	13.2±2.4	0.221	0.067	0.937	0.000	0.369	0.037
Trunk fat (%)	18.9±2.4	18.7±2.8	19.6±2.1	19.3±3.0	0.450	0.026	0.587	0.015	0.907	0.001
Abdomen LM (kg)	8.0±1.2	7.9±1.1	7.9±0.8	7.8±0.6	0.323	0.044	0.755	0.005	0.879	0.001
Abdomen FM (kg)	4.6±1.0	4.6±1.3	4.6±0.7	4.5±0.8	0.317	0.046	0.385	0.038	0.711	0.006
Abdomen fat (%)	6.7±0.9	6.6±1.2	6.6±0.6	6.4±0.6	0.357	0.029	0.286	0.057	0.544	0.041
Leg LM (kg)	16.2±1.8	16.2±1.7	16.4±1.6	16.5±2.0	0.841	0.002	0.426	0.032	0.820	0.002
Leg FM (kg)	8.1±2.6	8.2±2.5	8.5±1.6	8.7±1.8	0.222	0.067	0.603	0.014	0.536	0.018
Leg fatness (%)	11.8±2.7	11.9±2.7	12.2±1.6	12.8±2.7	0.133	0.100	0.168	0.093	0.268	0.055
TG (mmol·L^−1^)	1.0±0.5	1.0±0.5	1.2±0.4	0.9±0.4	0.065	0.160	0.537	0.022	0.073	0.152
TC (mmol·L^−1^)	4.3±0.8	4.3±0.4	4.2±0.8	4.2±0.5	0.766	0.004	0.081	0.144	0.786	0.003
HDL-C (mmol·L^−1^)	1.3±0.2	1.4±0.2	1.2±0.2	1.4±0.3	0.074	0.137	0.917	0.001	0.317	0.045
LDL-C (mmol·L^−1^)	2.4±0.8	2.3±0.3	2.3±0.6	2.3±0.3	0.476	0.023	0.093	0.135	0.846	0.002
TC/HDL-C	3.3±0.6	3.2±0.3	3.5±0.6	3.2±0.4	0.035	0.187	0.448	0.029	0.593	0.013
TG/HDL-C	0.8±0.5	0.8±0.5	1.0±0.4	0.7±0.4	0.027	0.223	0.618	0.014	0.067	0.158
Leptin (pg·ml^−1^)	479±26	500±33	484±19	481±29	0.300	0.049	0.739	0.006	0.171	0.083

*Values are expressed as means ± standard deviation. NORM, high-intensity interval training in normoxia; HYP, high-intensity interval training in normobaric hypoxia; $\overset{\cdot }{V}$O_2peak_, peak oxygen uptake, BMI: body mass index; LM, lean mass; FM, fat mass; BF, body fat (%); TG, triglycerides; TC, total cholesterol; HDL-C, high-density lipoprotein cholesterol; LDL-C, low-density lipoprotein cholesterol; TC/HDL-C, ratio of TC and HDL-C; TG/HDL-C, ratio of TG and HDL-C*.

Following the 5-wk HIIT intervention, there were no significant changes in whole-body and regional body composition in either NORM or HYP groups (*p* > 0.05). For the serum lipid profile, there were no significant changes in TG, TC, HDL-C, and LDL-C between pre- and post-intervention in both groups, while significant improvements in TC/HDL-C and TG/HDL-C were observed (*p* < 0.05) over time. Serum leptin was also unchanged between pre- and post-intervention in either group (*p* > 0.05).

## Discussion

To the best of our knowledge, this is the first study to investigate the effects of short-term hypoxic HIIT on cardiometabolic health in overweight Chinese sedentary young women. The main findings of this study were that 20 brief HIIT sessions over 5 weeks improved the cardiorespiratory fitness and blood lipids in both NORM and HYP groups. Moreover, the 5-wk HIIT carried out in HYP could boost further the training effect on the cardiorespiratory fitness, but not on the whole-body and regional body composition or serum lipid profile of the participants.

### Effects on cardiorespiratory fitness

$\overset{\cdot }{\text{V}}$O_2peak_ and peak O_2_ pulse are the variables frequently used in assessment of an individual's cardiorespiratory fitness in both research and clinical settings (Oliveira et al., 2009). In the present study, we found that the hypoxic HIIT led to a 2-fold increase in absolute $\overset{\cdot }{\text{V}}$O_2peak_ in comparison to that resulting from an identical normoxic training. Similar results were also found in peak O_2_ pulse (NORM: 13.4%; HYP: 25.9%). The current findings are in line with the results of previous studies where 3 to 4 weeks of hypoxic HIIT led to a further enhancement in aerobic capacity in athletes (Dufour et al., 2006; Czuba et al., 2011, 2013) as well in healthy young males (Bailey et al., 2000; Dufour et al., 2006). During the 5-wk hypoxic HIIT, despite the progressive rise in SpO_2_ of the HYP participants from the initial stage, possibly due to improved ventilation-perfusion inequality of the lung (Stoneham and Pethybridge, 1993), they were still suffering from hypoxia during the exercise. Nevertheless, the overall training impulse of the NORM group during the HIIT did not differ from that of the HYP group. Moreover, the duration of exercise at the intensity corresponding to ≥ 90% of HR_max_ tended to be longer in HYP group. The increase in duration of exercise at the aforementioned intensity from the initial to the final session of the HIIT accounted for 30.1% of the changes in absolute $\overset{\cdot }{\text{V}}$O_2peak_. This scenario suggests that the hypoxic HIIT might have overloaded the cardiovascular system additionally, and thus potentiated greater adaptation. It further provides supportive evidence that the short-term HIIT in a hypoxic environment is feasible in overweight and obese individuals to enhance aerobic fitness, and would result in an additive effect on cardioprotection when compared with the same training in normoxia.

It has been demonstrated that the activation of hematological adaptive mechanisms with intermittent hypoxic training requires the provision of sufficient training stimulus with the duration of hypoxic exercise last consecutively for 90 min for a single session (Rodriguez et al., 2000). In the present study, the additive increase in $\overset{\cdot }{\text{V}}$O_2peak_ resulting from the brief hypoxic HIIT regimen (≤ 20 min) is possibly associated with non-hematological adaptive mechanisms occurring in muscular and systemic portions. In fact, certain physiological adaptations in muscle tissues in response to hypoxic training, including increases in skeletal muscle mitochondrial density, capillary-to-fiber ratio, and fiber cross-sectional area, have been demonstrated to be larger than those resulting from traditional normoxic training in untrained individuals (Vogt et al., 2001; Zoll et al., 2006; Faiss et al., 2013b). Such discrepancy in training adaptations in muscles might be associated with an increase in hypoxia inducible factor-1α, which has been shown to be essential in regulating the cardiovascular and respiratory responses to hypoxia (Semenza, 2004). In addition, the additive increase in $\overset{\cdot }{\text{V}}$O_2peak_ in the HYP group is concomitant with a greater peak O_2_ pulse. The specific adaptations in the cardiovascular system to hypoxic training, including increased stroke volume, might lead to more effective systemic circulation for optimizing muscle oxygenation (Czuba et al., 2011).

### HYP on blood lipids and body composition

It is known that exposure to hypoxia could facilitate lipid metabolism through the transcriptional coactivator peroxisome proliferator-activated receptor-1, which is triggered by the hypoxia inducible factor-1 (Zoll et al., 2006; Millet et al., 2010). Modifications of blood lipid profile with short-term hypoxic training have been demonstrated in untrained individuals. Haufe et al. (2008) reported that 4-wk hypoxic training in untrained healthy men reduced blood TG to a greater extent in comparison to training in normoxia. Such beneficial effects of hypoxic training on blood lipid profile were also observed in healthy normal-weight males following a 10-day intervention of normobaric hypoxic (FIO_2_ = 14%) confinement (Debevec et al., 2014). However, the 5-wk hypoxic HIIT in the present study did not lead to significant additive improvements in serum lipids in the HYP group. The current findings were consistent with those found previously in overweight or obese subjects following a 4-wk moderate-intensity treadmill training regimen under hypoxia (Wiesner et al., 2010). The comparable post-intervention profile of serum lipids between NORM and HYP groups was in accordance with the previous notion that short-term moderate hypoxic training does not cause significant additive lipolytic effect to that invoked by normoxic training, specifically, when the body mass of participants remains constant and their diet and physical activity levels are controlled (Bailey et al., 2000). Differences in training mode and regimens (low-to-moderate aerobic training vs. HIIT), the body fat status of participants (normal vs. overweight vs. obese), and their dietary control may definitely cause contradictory findings.

Improved body composition has been reported during or after acute high altitude exposure (Lippl et al., 2010). Combining hypoxic exposure with exercise training has also been reported to potentiate weight loss in overweight and obese individuals (Netzer et al., 2008; Wiesner et al., 2010; Kong et al., 2014). For instance, low-intensity exercise performed three times per week for 90 min with normobaric hypoxia (FIO_2_ = 15%) for 8 weeks could lead to greater body weight reduction in obese individuals than resulted from training under sham hypoxia (Netzer et al., 2008). Similar results were also noted from a relatively brief training regimen which was composed of three sessions of 60 min hypoxic endurance exercises per week for 4 weeks in overweight/obese subjects (Wiesner et al., 2010). However, greater improvement was not observed in body composition and fasting serum leptin level in our HYP participants participating in the 5-wk hypoxic HIIT compared with the NORM group. The current training outcomes, in contrast to the previous findings, might have been derived from differences in training mode and intensities which have been shown to play essential roles in regulating free-fatty acid mobilization (Romijn et al., 1993). It is well known that blood flow increases in subcutaneous adipose tissue in response to low-intensity exercise, and plateaus when exercise intensity is further increased (Heinonen et al., 2012). This plateau in adipose tissue blood flow during high-intensity exercise is considered an important mechanism for decreasing free fatty acid mobilization during the exercise (Romijn et al., 1993), and alternatively, increasing the contribution of aerobic/anaerobic glycolysis to ATP generation. Recently, it was found that adipose tissue blood flow is further reduced during exercise with hypoxic breathing, possibly due to hypoxia-triggered enhanced sympathetic vasoconstriction in adipose tissue vasculature (Heinonen et al., 2014). Although the present study did not examine fat metabolism during the 5-wk HIIT, it is reasonable to postulate that a marked increase in fat metabolism in the HYP group in comparison to that of NORM group is unlikely to occur during the intervention period. In fact, the beneficial effects of hypoxic training on body composition in most previous studies were with low-to-moderate intensity continuous exercise (Haufe et al., 2008; Netzer et al., 2008; Wiesner et al., 2010; Kong et al., 2014). Similar findings regarding body fat control with HIIT under hypoxia either in healthy or obese individuals are limited. In addition, increased serum levels of the satiety-signaling hormone leptin and the resultant anorexia response are generally associated with altitude sojourn or hypoxic training (Shukla et al., 2005). Nevertheless, the unchanged serum leptin found in the HYP group following the 5-wk hypoxic HIIT is in line with previous findings in short-term hypoxia exposure (Debevec et al., 2014) or hypoxic training (Haufe et al., 2008) in healthy individuals, and support the notion that hypoxia does not necessarily always affect one's serum leptin and associated appetite regulation (Debevec et al., 2014). Apart from potential intensity difference which may partly explain the contrary findings in body fat reduction, participants' initial adiposity level may also impact changes in body fat resulting from an exercise intervention (Forbes, 2000). It is known that participants with a higher BMI appear to have more fat loss after an intervention compared with those possessing a lower BMI (Hansen et al., 2001). In the present study, the initial BMI (~25 kg·m^−2^) of the Chinese female participants appeared to be lower than that (~30 kg·m^−2^) reported in previous studies (Haufe et al., 2008; Netzer et al., 2008; Wiesner et al., 2010; Kong et al., 2014). It is therefore conceivable to consider the relative low initial BMI as an apparent disadvantage in revealing the impact of the 5-wk HIIT on body fat loss.

### Limitations

In the present study, there are several caveats which deserve discussion. First, the habitual calorie intake and energy expenditure of participants in this study were estimated based on the self-reported dietary intake, and the data of activity monitoring, respectively, recorded 3 days per week. Moreover, the lead-in period for the dietary and physical activity assessments was only 1 week in advance of the intervention. Although our findings provide reasonable information regarding the daily energy intake and output during the intervention, further interpretation of the current findings is therefore limited by the potential estimation errors. In avoidance of the potential errors, participants accommodating in a hypoxic training camp with strict control in nutritional intake and physical activity is recommended. Such a maneuver had been successfully implemented in our previous study (Kong et al., 2014). Secondly, a gradual increase in SpO_2_, possibly due to ventilation-perfusion adaptation in the lungs (Stoneham and Pethybridge, 1993), demonstrated the progressive attenuation of hypoxic stress in participants during the intervention period. It is reasonable to hypothesize the further improvement would be observed in the resultant aerobic capacity, as well as the body composition and serum lipid profile in HYP participants if the initial SpO_2_ and associated hypoxic stress could be maintained throughout the intervention period. These points await further investigation. Moreover, the improvements in the cardiorespiratory fitness and serum lipids resulting from HIIT under mild hypoxia could possibly be augmented by increasing the severity of hypoxia. However, more severe hypoxia might not be well tolerated by all individuals. It might concomitantly induce physiological detrimental effects on bodily function including inflammatory responses in adipose tissue of obese persons (Heinonen et al., 2016). A balance of these factors appears to be the prerequisite for achieving potential health benefits of hypoxia on humans. Lastly, as Chinese population generally possess a lower level of adiposity than their European counterpart (Lesser et al., 2013), such ethnical difference in body fat accumulation and distribution would lead to inconsistent hypoxic HIIT effects on body composition.

## Conclusion

Using a randomized, double-blind, placebo-controlled design in overweight Chinese young women, the present study demonstrates that the specific 5-wk HIIT regimen could improve cardiorespiratory fitness and blood lipids in the participants in both NORM and HYP groups. Nevertheless, the additive effect of the HIIT under mild hypoxia (FIO_2_ = 0.15) only appears to enhance cardiorespiratory fitness, but not the whole-body and regional body composition, or serum lipid profile.

## Ethics statement

The ethical group in the University of Macau. Overweight healthy young females were recruited to participate in the study through local advertisements. After the initial screening, the qualified participants were informed verbally and in writing of the experimental procedures and associated risks in detail, all participants gave their written consent to participate in the study. During the intervention, all participants can withdraw from the study at any time without any reasons. In order to avoid any unexpected risks, a physician was in attendance during all tests and training sessions associated with the study.

## Author contributions

The contributions of the authors were as follows: research design: ZK, QS, JN, TT, and YH; data collection: LY, LS, and YH; data analysis and interpretion: ZK, QS, JN, and TT; manuscript drafting: ZK, QS, and LS; manuscript revision: JN, TT, LY, and YH.

## Funding

This study was supported by a research grant received by ZK from the University of Macau (MYRG027(Y1-L1)FED11-KZW), which made no contribution toward research design, data collection or decision to publish.

### Conflict of interest statement

The authors declare that the research was conducted in the absence of any commercial or financial relationships that could be construed as a potential conflict of interest.

## Abbreviations

BF: body fat
BMI: body mass index
HDL-C: high-density lipoprotein cholesterol
FM: fat mass
HIIT: high-intensity interval training
HR: heart rate
HYP: normobaric hypoxia
LDL-C: low-density lipoprotein cholesterol
LM: lean mass
NORM: normoxia
SpO_2_: peripheral oxygen saturation
TC: total cholesterol
TG: triglycides
TC/HDL-C: ratio of TC and HDL-C
TG/HDL-C: ratio of TG and HDL-C
$\overset{\cdot }{\text{V}}$O_2_: oxygen consumption
$\overset{\cdot }{\text{V}}$O_2peak_: peak oxygen uptake.
